# Supplementary material for: Fast and simple tool for the quantification of biofilm-embedded cells sub-populations from fluorescent microscopic images
Source: PLoS One. 2018 May 1;13(5):e0193267. doi: 10.1371/journal.pone.0193267 (PMC5929543; doi:10.1371/journal.pone.0193267)

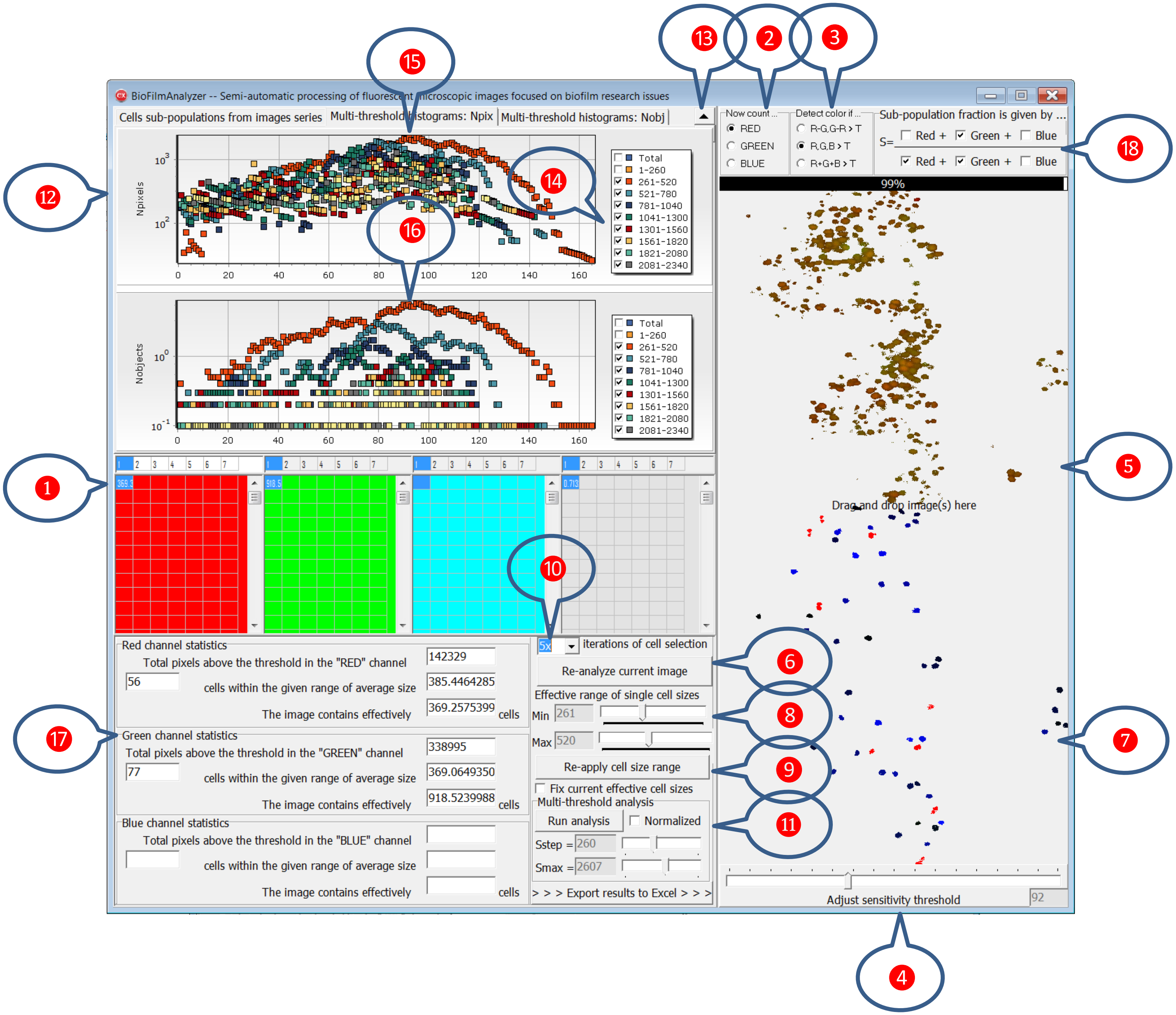
BioFilmAnalyzer -- Semi-automatic processing of fluorescent microscopic images focused on biofilm research issues
Cells sub-populations from images series
Multi-threshold histograms: Npix
Multi-threshold histograms: Nobj
Now count ...
RED
GREEN
BLUE
Detect color if ...
Sub-population fraction is given by ...
Npixels
Nobjects
Total
1-260
261-520
521-780
781-1040
1041-1300
1301-1560
1561-1820
1821-2080
2081-2340
Drag and drop image(s) here
Red channel statistics
Total pixels above the threshold in the "RED" channel
cells within the given range of average size
The image contains effectively
cells
Green channel statistics
Total pixels above the threshold in the "GREEN" channel
Blue channel statistics
Total pixels above the threshold in the "BLUE" channel
iterations of cell selection
Re-analyze current image
Effective range of single cell sizes
Min
Max
Re-apply cell size range
Fix current effective cell sizes
Multi-threshold analysis
Run analysis
Normalized
Sstep =
Smax =
> > > Export results to Excel > > >
Adjust sensitivity threshold
1
2
3
4
5
6
7
8
9
10
11
12
13
14
15
16
17
18

1 Start here. Fill the column headers with some parameters characterizing your experimental setting, e.g. drug concentrations. Choose which color staining you would like to analyze first and the initial cell where to paste the cell count data, e.g. the upper left corner of the red table like in this example. Please note that choosing an active cell in another table (e.g. green) will automatically adjust the choice of the color in 2.

3 Choose the selection rule. By default, the color channel rule is applied, i.e. the point is considered as red-stained when the intensity of the red channel exceeds T. Alternatively, you can select rules based on either differential or overall (normalized) intensities exceeding T which can be tuned between 0 and 255 using 4.

5 Now try to process your sample image by drag&drop in this area. The processing will start instantly, indicated by the progress bar just above the image. As it is completed, you will see only parts of your image that satisfy the rule 3 for chosen channel 2 and sensitivity threshold 4. If you are not satisfied with the choice of the parameters, you can always adjust them by 2 3 4 and repeat the image processing using 6. At any given time you can switch between the processed and the original image by single-click anywhere in 5.

7 Here you will see the results of your image segmentation. The segmentation procedure selects all isolated objects, e.g. cells from the processed image above. You should aim at selecting those fragments that represent typical single cells. For that you can specify the range of the typical cell sizes by 8 and repeat the segmentation procedure using 9. Image segmentation is generally an iterative procedure, with the number of iterations always being a certain compromise between accuracy and performance. While the default settings work fine in most typical scenarios, for some complex-shaped objects such as stem cells you may eventually see that the segmentation is inaccurate and thus need to increase the number of iterations by 10. Alternatively, for simple-shaped cells you can decrease it this way gaining better performance.

Please note that for larger T values the same cells are usually represented by objects of smaller sizes. One may find the proper balance between the sensitivity threshold T and the single cell size range empirically in a trial-and-error fashion. However, a more scientific approach would be to learn a bit more about the statistical properties of your images before making a final choice. For that, set up size resolution and maximum size of objects of interest in and run multi-threshold analysis 11. Shortly the histograms of object sizes and counts for all possible T values will be depicted in 12. Expand histograms by 13 to see also object count. Choose the size ranges of interest by 14, in most cases excluding very small object sizes, and find the histogram maximum 15. In most cases, it should correspond to the maximum 16 in the object count plot. By clicking on the histogram point, the chosen threshold T and single size range will be automatically copied to 4 and 8. Try whether the chosen settings work fine in your case by 6. Eventually, choosing a wrong peak may lead to the selection of other objects than single cells, e.g. cell clusters or noise speckles, then try with another one. If you feel that the despite of all efforts the range-based selection is not accurate enough, you can remove redundant entries by selecting them directly in 7 (here selected items are shown in red) followed by a double-click anywhere in 7. Please note that you should not aim at selecting all or nearly all cells at this step. They could be just a few but should be of the representative size of single cells.

17 Results of single image processing are summarized in these panels, separately for each color channel. Briefly, the effective number of cells is obtained as the total area above the threshold in 5 divided by the effective cell size that in turn is determined from 7. Of note, non-viable cells often exhibit smaller sizes compared to viable cells that may require using different effective cell size ranges 8 for different color channels.

18 In order to calculate the relative fractions of sub-populations, you can define the rule according to your experimental setting. The results will appear in the sub-populations table and will be automatically adjusted every time you change the sub-population rule.

Once you are satisfied with the results for a single or few representative images, you can turn to **automatic processing of image series,** given that they are rather homogeneous. Hide the histograms by 13 and keep all other settings that worked well for your sample image(s). Choose the starting cell for the series in the respective table for given color 1 and drag&drop multiply selected images into 5. The entire series of up to 100 images will be processed consecutively, with the results filling the

chosen column in 1 downwards from chosen starting point. Repeat the same procedure for other color channel(s) if necessary by choosing corresponding target cells in the color tables.

For **several series of experiments with changing parameter,** e.g. drug concentration, choose each column for a series of measurements obtained for each concentration. When several columns are filled, the results will also appear in the graphical form in 19 including data points and trend lines. Eventually missing data in 1 may indicate that some of the images in your series lack statistics of cells of respective color to estimate the effective single cell size. In this case, given that the image series is rather homogeneous, you may like to fix the effective cell size obtained from representative image(s) by 20 and repeat calculations for missing data.

You can also specify explicitly which data you would like to display within 19 where the sub-population fraction is determined by the rule 18. For further statistical analysis and dissemination of the results, you can export the data accumulated so far in all four tables, including color channels data and sub-population data, as an Excel workbook at any time using 0. Note that each export procedure creates a new instance of Excel that has to be closed manually after saving the necessary data, especially on intermediate steps in order to save memory.

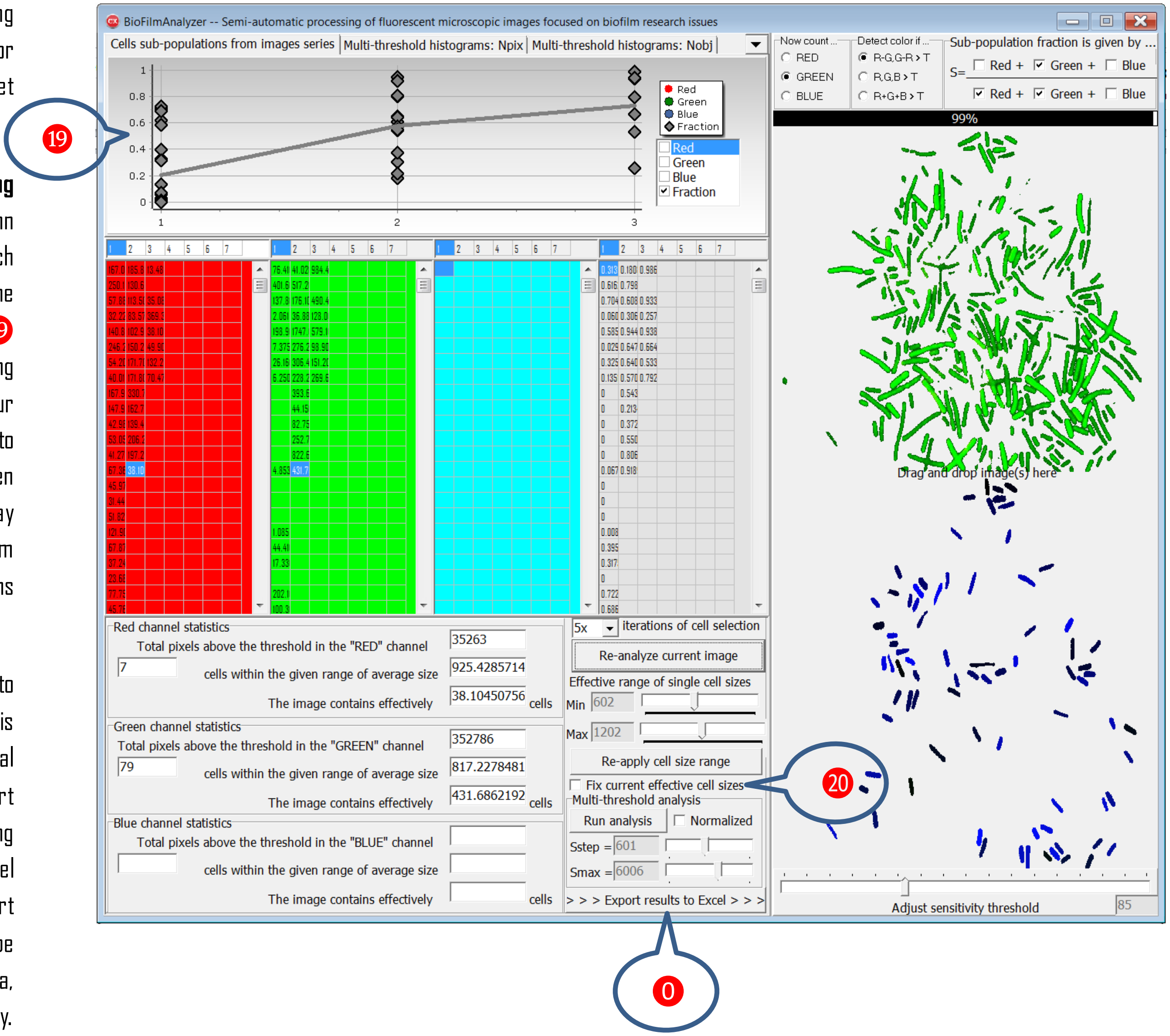

Supplement: S2 File — Can be downloaded from https://bitbucket.org/rogex/biofilmanalyzer/downloads/. (PDF) [file pone.0193267.s002.pdf]
